# Alterations of Mass Density and 3D Osteocyte Lacunar Properties in Bisphosphonate-Related Osteonecrotic Human Jaw Bone, a Synchrotron µCT Study

**DOI:** 10.1371/journal.pone.0088481

**Published:** 2014-02-21

**Authors:** Bernhard Hesse, Max Langer, Peter Varga, Alexandra Pacureanu, Pei Dong, Susanne Schrof, Nils Männicke, Heikki Suhonen, Cecile Olivier, Peter Maurer, Galateia J. Kazakia, Kay Raum, Francoise Peyrin

**Affiliations:** 1 European Synchrotron Radiation Facility, Grenoble, France; 2 Berlin-Brandenburg School for Regenerative Therapies & Julius Wolff Institut, Charité, Universitätsmedizin Berlin, Germany; 3 Université de Lyon, CREATIS, CNRS UMR5220, INSA-Lyon, Lyon, France; 4 Centre for Image Analysis and Science for Life Laboratory, Uppsala University, Uppsala, Sweden; 5 Klinik für Mund-, Kiefer- und Gesichtschirurgie, Klinikum Bremerhaven-Reinkenheide, Kiel, Germany; 6 Department of Radiology and Biomedical Imaging, University of California San Francisco, San Francisco, California, United States of America; Faculté de médecine de Nantes, France

## Abstract

Osteonecrosis of the jaw, in association with bisphosphonates (BRONJ) used for treating osteoporosis or cancer, is a severe and most often irreversible side effect whose underlying pathophysiological mechanisms remain largely unknown. Osteocytes are involved in bone remodeling and mineralization where they orchestrate the delicate equilibrium between osteoclast and osteoblast activity and through the active process called osteocytic osteolysis. Here, we hypothesized that (i) changes of the mineralized tissue matrix play a substantial role in the pathogenesis of BRONJ, and (ii) the osteocyte lacunar morphology is altered in BRONJ. Synchrotron µCT with phase contrast is an appropriate tool for assessing both the 3D morphology of the osteocyte lacunae and the bone matrix mass density. Here, we used this technique to investigate the mass density distribution and 3D osteocyte lacunar properties at the sub-micrometer scale in human bone samples from the jaw, femur and tibia. First, we compared healthy human jaw bone to human tibia and femur in order to assess the specific differences and address potential explanations of why the jaw bone is exclusively targeted by the necrosis as a side effect of BP treatment. Second, we investigated the differences between BRONJ and control jaw bone samples to detect potential differences which could aid an improved understanding of the course of BRONJ. We found that the apparent mass density of jaw bone was significantly smaller compared to that of tibia, consistent with a higher bone turnover in the jaw bone. The variance of the lacunar volume distribution was significantly different depending on the anatomical site. The comparison between BRONJ and control jaw specimens revealed no significant increase in mineralization after BP. We found a significant decrease in osteocyte-lacunar density in the BRONJ group compared to the control jaw. Interestingly, the osteocyte-lacunar volume distribution was not altered after BP treatment.

## Introduction

Under healthy conditions bone undergoes continuous remodeling to adapt to spatially and temporarily variable demands, through a delicate equilibrium between resorption and formation, which is performed by osteoclast and osteoblast cells, respectively.

Bisphosphonates (BP), which are commonly prescribed in the treatment of osteoporosis and bone metastasis, have been shown to reduce significantly the risk of fracture [1], [2]. The action of BP relies on the reduction of bone resorption by inhibiting osteoclast activity. However, a severe and most often irreversible adverse effect of high-dosage BP treatment is the potential occurrence of osteonecrosis of the jaw [3]–[7]. Although multiple hypotheses have been formulated recently, the underlying pathophysiological mechanisms of bisphosphonate-related osteonecrosis of the jaw (BRONJ) are still not completely understood [8]–[11].

Bone remodeling results in a heterogeneous distribution of mineralized tissue units, with variable degrees of mineralization. This heterogeneity can be assessed from the bone mineralization density distribution by using techniques such as quantitative backscattered electron imaging (qBEI) [12]–[14], microradiography [15], or synchrotron radiation micro-CT [14], [16]. The degree of mineralization of bone is a quality factor that influences the mechanical properties of bone [17].

Osteoclast and osteoblast activity is thought to be orchestrated by osteocytes, which are the most abundant type of bone cell and form a well-distributed network within the mineralized matrix [18]. These cells reside in cavities called lacunae measuring several hundreds of µm^3^ in volume, and are interconnected through cell dendrites extending in tiny canals called canaliculi. The canalicular diameter of human bone has been reported to be within the range between 200 and 900 nm [19].

Osteocyte activity is thought to be stimulated by biological and mechanical signals [20]. The morphology of the lacuno-canalicular network (LCN) is believed to be related to the mechanosensation and mechanotransduction processes of osteocytes [21]–[31]. Furthermore, the LCN ensures the transport of cellular waste and nutrients [23]. The LCN has also been reported to be essential for micro-crack repair by triggering bone remodeling [32]. In addition to their mechanical function, it is hypothesized that osteocytes regulate mineral metabolism, e.g. bone phosphate metabolism [33], [34].

It was recently shown in a murine lactating model that not only osteoclasts are able to resorb bone matrix, but also that osteocytes remodel their peri-lacunar and peri-canalicular matrix [35]. Alterations in lacunar size have also been observed in response to changes of the mechanical environment, for example enlarged lacunae were reported in mice following space flight [36], or after glucocorticoid treatment [37]. In ovariectomized rats, both lacunar size and density were found to be altered in newly-formed bone after antiresorptive and anabolic pharmaceutical treatment [38].

So far, investigation of the three-dimensional (3D) structure of the LCN has been limited by the imaging techniques available [21], [39]. Synchrotron radiation micro-computed tomography (SR µCT) enables 3D imaging of bone tissue at the cellular length scale and has been shown to be an appropriate tool for investigating 3D lacunar morphology [40]–[43]. At the sub-micron resolution, SR µCT enables 3D imaging of the LCN with a large field of view [44], [45]. Synchrotron X-ray nano-CT with phase contrast, which provides sensitivity to the mass density variations that is several orders of magnitude higher than conventional attenuation contrast SRµCT, was recently used to investigate the bone LCN and the 3D collagen orientation at the nanometer length-scale [46], [47].

However, very limited data is currently available at the sub-micron length scale for human jaw bone, both in terms of the distribution of osteocyte lacunae and in terms of mass density distribution [48].

In the present study, we investigated the differences in lacunar morphology and peri-lacunar tissue properties at the sub-micrometer length scale in human jaw bone tissue samples obtained from both healthy subjects and patients suffering from BRONJ. Additionally, cortical bone samples collected from the femur and tibia of donors not treated with BP were analyzed and compared to BRONJ and healthy control jaw bone samples. All investigations were based on SR µCT using phase contrast images measured with a 350 nm isotropic pixel size. Coupled with phase retrieval, this simultaneously provides information about the 3D distribution of the osteocyte lacunae and the local mass density of bone [46]. In order to quantify the mass density distribution, which is closely related to the bone mineral density distribution (BMDD), we used indices similar to those introduced for BMDD [13]. We hypothesized that (i) the bone turnover of the healthy human jaw bone, assessed by analyzing the mass density distribution, would be increased in comparison to the other anatomical sites and (ii) that extracellular matrix density and lacunar volumes of samples originating from patients suffering from BRONJ would be altered in order to compensate for the mineral homeostasis disturbed by the inhibited osteoclast activity. We therefore reported and compared the osteocyte lacunae volume distribution and the spatial arrangement of lacunae, as well as descriptors for the mass density distribution of the peri-lacunar tissue.

## Materials and Methods

### Ethics Statement

Ethical approval for the jaw samples was granted by the Ärztekammer Bremen (Studien-Nr. 310). All donors signed an informed consent form. The present study also involved the use of cadaver specimens.

Ethical approval for the femur samples was granted by the Ethical Commission of the Medical University of Vienna, see [49]. Ethical approval for the tibia was granted by the University of California, San Francisco Committee on Human Research, see [50].

### Specimen Preparation

Nineteen human jaw bone sections (blocks of about 1–3 mm^3^ in size) were extracted from 12 female and 7 male donors, of whom 8 female and 2 male donors were suffering from BRONJ. The healthy control samples were from debris obtained during tooth removal. The BRONJ samples were obtained from surgeries necessary for the treatment of the necrosis. Furthermore, 7 cadaver specimens originating from the human femoral midshaft and 3 cadaver specimens originating from the human tibia midshaft of other donors were included in the present study. Detailed information on gender, donor age, anatomical origin, and BP treatment can be found in Table 1.

**Table 1:** Sample details such as age, gender, anatomical region, BP treatment and duration of the BP treatment are listed.

Internal samplename	Site	Gender	Region	Age	BP treatment	Duration of BP treatment	Underlying diagnosis
tib29	tibia	male	midshaft	29	–		
tib56	tibia	male	midshaft	56	–		
tib88	tibia	male	midshaft	88	–		
fem5RF68	femur	female	midshaft	68	–		
fem15RF66	femur	female	midshaft	66	–		
fem11LF64	femur	female	midshaft	64	–		
fem11LF87	femur	female	midshaft	87	–		
fem1LF70	femur	female	midshaft	70	–		
fem2RM60	femur	male	midshaft	60	–		
fem1LM71	femur	male	midshaft	71	–		
jaw3wk5	jaw (BRONJ)	female	37	n. k.	Z	>1 year	n. k.
jaw1wk4	jaw (BRONJ)	female	35	75	Z	13 months	Mammary-carcinoma
jaw1wk5B	jaw (BRONJ)	female	n. k.	70	Z	16 months	Plasmacytom
jaw2wk2A	jaw (BRONJ)	female	45	70	A	17 months	Osteoporosis
jaw3wk1A	jaw (BRONJ)	female	13	72	A	10 years	Osteoporosis
jaw2wk36A	jaw (BRONJ)	female	36/37	74	A	19 months	Osteoporosis
jaw2wk1	jaw (BRONJ)	female	15	44	Z	1 year	Mammary-Carcinoma
jaw3wk4	jaw (BRONJ)	female	45	n. k.	Z	2 years	n. k.
jaw1mk4	jaw (BRONJ)	male	15	84	Z	10 years	Prostate-carcinoma
jaw2mk1	jaw (BRONJ)	male	16/17	81	Z	14 months	Multiple myeloma
jaw2mg2	jaw (control)	male	36	44	–		
jaw2mg6A	jaw (control)	male	48	19	–		
jaw2mg3	jaw (control)	male	48	54	–		
jaw2mg4	jaw (control)	male	47	42	–		
jaw3mg6	jaw (control)	male	33/34	27	–		
jaw2wg5	jaw (control)	female	42	42	–		
jaw2wg3B	jaw (control)	female	17	40	–		
jaw2wg1	jaw (control)	female	16	68	–		
jaw2wg4	jaw (control)	female	42	47	–		

‘Z’: intravenous administration of Zoledronate, ‘A’: oral administration of Alendronate, ‘n.k.’: not known.

The femora were collected and prepared as described previously [49]. After removal, the jaw bone sections were embedded in Tissue-Tek® O.C.T.™ (Sakura Fintec Europe B.V., Alphen aan den Rijn, Netherlands) solution and stored frozen at −20°C until further processing. Following thawing, the specimens were drilled down to a diameter of about 0.5 mm using a high precision lathe [51]. For the BRONJ samples, this sub-volume was selected from a region in which no necrotic tissue had been observed. The cut bone samples were fixed in 70% ethanol for transport. Tibia bone cores were machined with a coring tool and precision circular saw to a length of 4 mm and diameter of 4 mm, similar to the sample preparation described in [50]. Then, to fit the field of view of the imaging setup, the tibia samples were cut from cortical bone (diameter: 500 µm, height: 1 mm) using a high precision drilling machine. About 12 hours before imaging, the samples were placed inside the measurement hutch in order to allow adjustment to humidity and temperature.

### Synchrotron Radiation Phase Contrast µCT

The SR µCT data were obtained at ESRF (European Synchrotron Radiation Facility, Grenoble, France) at beamline ID22NI. The X-ray beam was focused using Kirkpatrick-Baez reflective optics [46]. The scans used in the present study were performed for each sample by collecting 1201 projections, each with 0.2 s exposure time, over a total range of 360°. The energy was set to 16.874 keV, and the sample-detector distance was 282 mm, resulting in a (350 nm)^3^ isotropic voxel size in the reconstructed image. Due to the coherence of the synchrotron source, the intensity of the recorded radiograph includes phase contrast [52], [53]. Reconstruction was performed using Paganin’s method [54], coupled to the conventional filtered back projection algorithm.

**Figure 1:**
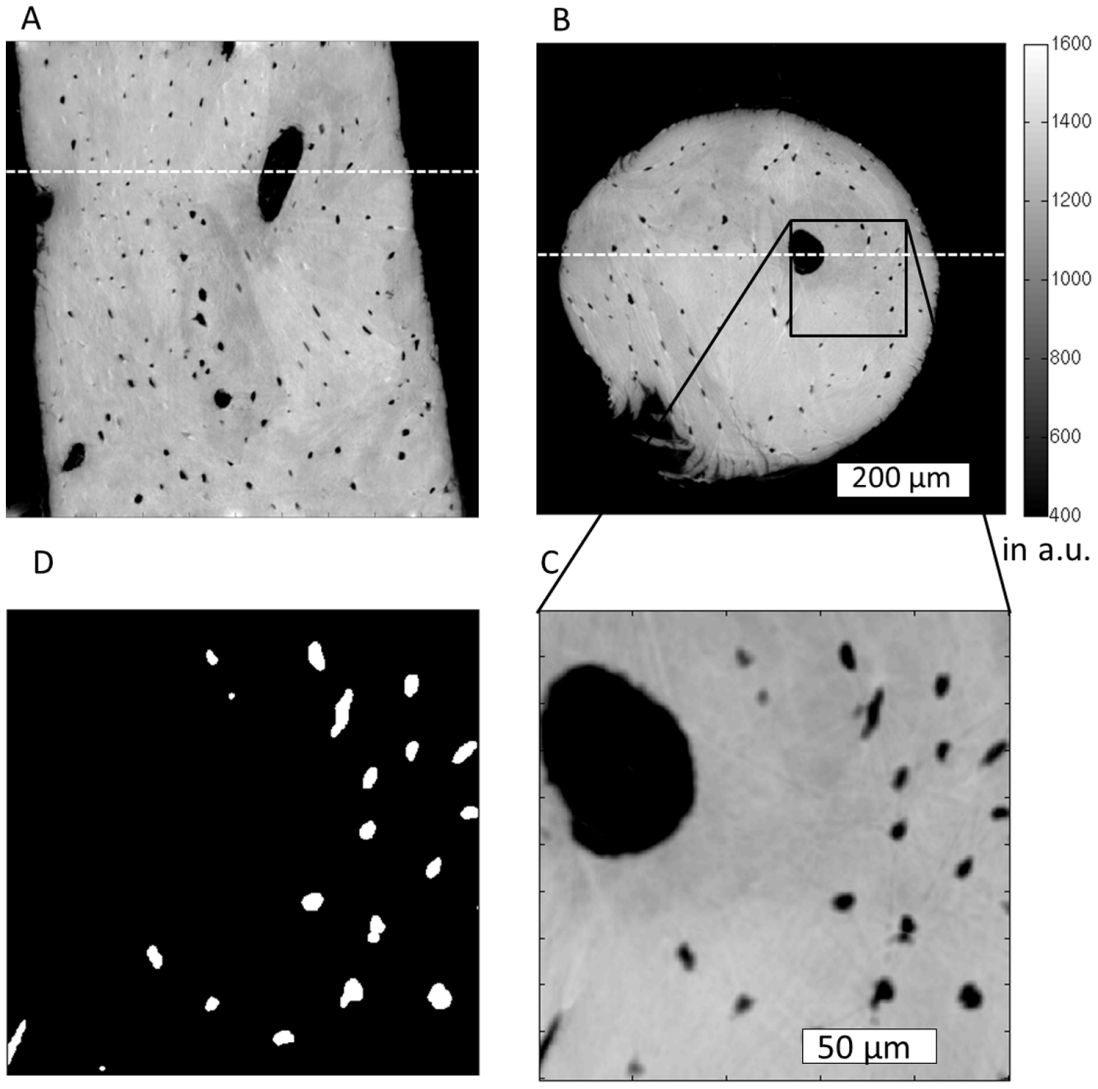
Slides of a reconstructed volume corresponding to a control jaw specimen are shown in A (x-y-plane) and B (x-z-plane). The white dashed lines in A and B indicate where A is located in B and vice versa. C shows a minimum intensity projection, the projection range is 30 pixels (10.5 µm). D shows the lacunae mask corresponding to C, also in the form of an intensity projection (z-range = 30 pixels). Color bar in mass density of a.u.

In the Paganin method, the phase is retrieved by simply assuming a linear relationship between the absorption index (β) and the refractive index decrement (δ). For cortical bone (ICRU-44), the δ/β (delta/beta) ratio at the given energy was set to 199 based on the XOP software [55]. The high ratio of delta/beta demonstrates the higher sensitivity for imaging the phase (δ) compared to imaging the attenuation (β). The reconstructed 3D image made of 2048^3^ voxels corresponds to a map of the refractive indices stored in units of 2π/λ, with λ being the wavelength of the X-ray beam (here λ = 0.0735 nm). This map is linearly related to mass density [46] which was shown to be associated with the degree of mineralization [56]. The spatial resolution with these settings allowed an easy distinction of osteocyte lacunae and larger pores from the mineralized tissue matrix (Fig. 1A–C). However, the canalicular network could be not resolved.

### Image Segmentation

**Figure 2:**
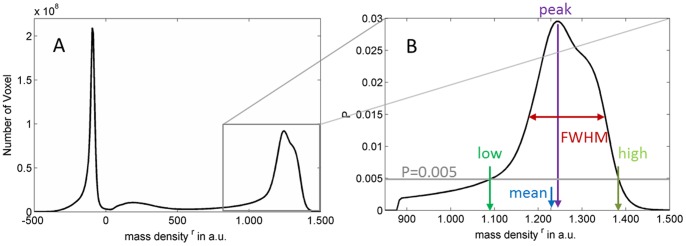
In (A) a relative mass density distribution is shown. The peak at around −100 is due to the air outside the sample, and the broad peak at about 200 is due to the glue used to attach the sample that managed to travel up the sample. In (B) the 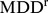 after segmentation is shown, and the parameters derived from it are indicated.

**Figure 3:**
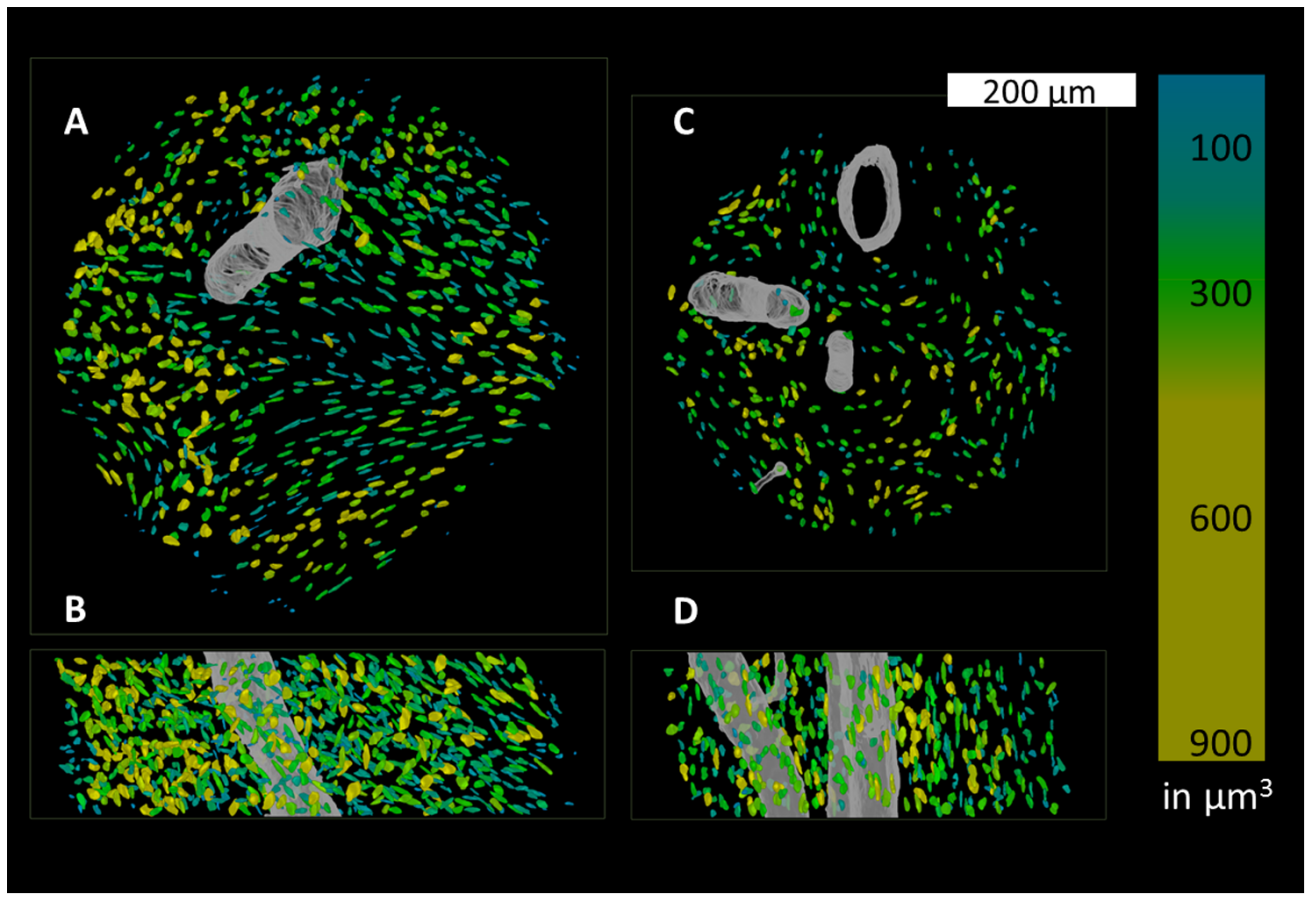
Volume renderings of two subsections originating from the jaw (A, B) and the femur (C, D. A and C show the lacunae and canals from a “top-down” perspective while B and D are shown from a “front-on” perspective. The volumes of the lacunae are color-coded in units of µm^3^.

In order to segment osteocyte lacunae inside the bone tissue volume (BV), the histogram of the whole 3D image was computed (Fig. 2A). A threshold was determined for each image using the multi-class Otsu’s method in the open-source software ITK (Kitware) [57] to separate mineralized tissue from non-mineralized pores. The resulting binary image was then labeled using a 3D connected component (CC) analysis method [41]. Objects smaller than 50 µm^3^ or larger than 1000 µm^3^ were considered not to be lacunae and were excluded from further analysis (Fig. 3).

### Extraction of Quantitative Parameters

The 3D image of each sample was virtually divided along the sample length into 3 equal-sized volumes of interest (VOI). The lacunae connected to the border, which could be truncated, were removed to avoid including bias in the analysis. Bone volume (BV) was considered as the entire mineralized tissue excluding osteocyte lacunae and other pores. The lacunae were segmented as described above and their volumes were computed for each VOI. The median (Lc.V_med_) and the variance (Lc.V_var_) were extracted from the histogram of the lacunar volumes. Lacunar porosity was derived as the ratio of the total volume of all lacunae to the bone volume (Lc.TV/BV) and the lacun 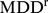 ae density was defined as the number of lacunae per bone volume (N.Lc/BV). Furthermore, for each VOI, the distance within which 50% of the mineralized bone tissue is located with respect to the closest lacunar surface (Lc.Dist_50_) was computed as the median of the Euclidean distance transform of the bone tissue [43].

Additionally, we used the reconstructed complex refractive index distribution, which is linearly related to the mass density, to compute the apparent mass density distribution 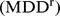 of each sample (Fig. 2B). The 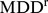 was calculated within the BV domain and was normalized by its area under the curve. Since the reconstructed complex refractive index might be biased due to the constant delta over beta ratio used in the Paganin phase retrieval [58], absolute values of mass density could not be retrieved and hereinafter the superscript *r* denotes relative values for all mass density parameters.

However, since the different bone samples can be considered comparable in terms of size and composition, this allows quantitative comparison of the relative difference in mass density between the bone samples.

Following the well-established approach for the description of BMDD by Roschger et al. [13], five parameters were extracted from the 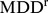, i.e. 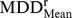 (the mean relative mass density within the evaluated distribution), 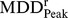 (the most frequent relative mass density value), 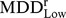 and 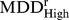 (the 0.5^th^ and 99.5^th^ percentiles), and 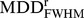 (the full width at half maxima of the distribution). The threshold of P = 0.005 was arbitrarily chosen and is a compromise between maintaining good sensitivity for low and high values in the 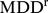 and minimizing potential artifacts using the partial volume effect for 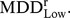


Eventually, the vascular porosity was estimated after coarsening the segmented volumes by a factor of five and cleaning the volumes from objects smaller than 1600 voxel (8575 µm^3^). The canal volume (Ca.V) was quantified using voxel counting and canal surface (Ca.S) was determined from the number of voxel located within one voxel Euclidean distance to the pore boundary. The following parameters were quantified: ratio of canal volume to bone volume (Ca.V/BV), ratio of canal surface to bone volume (Ca.S/BV) and ratio of canal surface to canal volume (Ca.S/Ca.V).

All post-processing was done using MATLAB 2012a (The MathWorks Inc., Natick, MA, USA).

### Statistical Testing

All statistical analyses were performed using the statistics toolbox in MATLAB. The normality of the distributions of each investigated parameter was determined by the Jarque-Bera test [59]. Differences with respect to anatomical sites, healthy and BRONJ groups were assessed by analyses of variance (ANOVA), followed by post hoc multiple comparison Bonferroni tests. The sample size did not allow a robust analysis of the effects of age and gender. All statistical results were considered significant for *p*<0.05.

## Results

**Figure 4:**
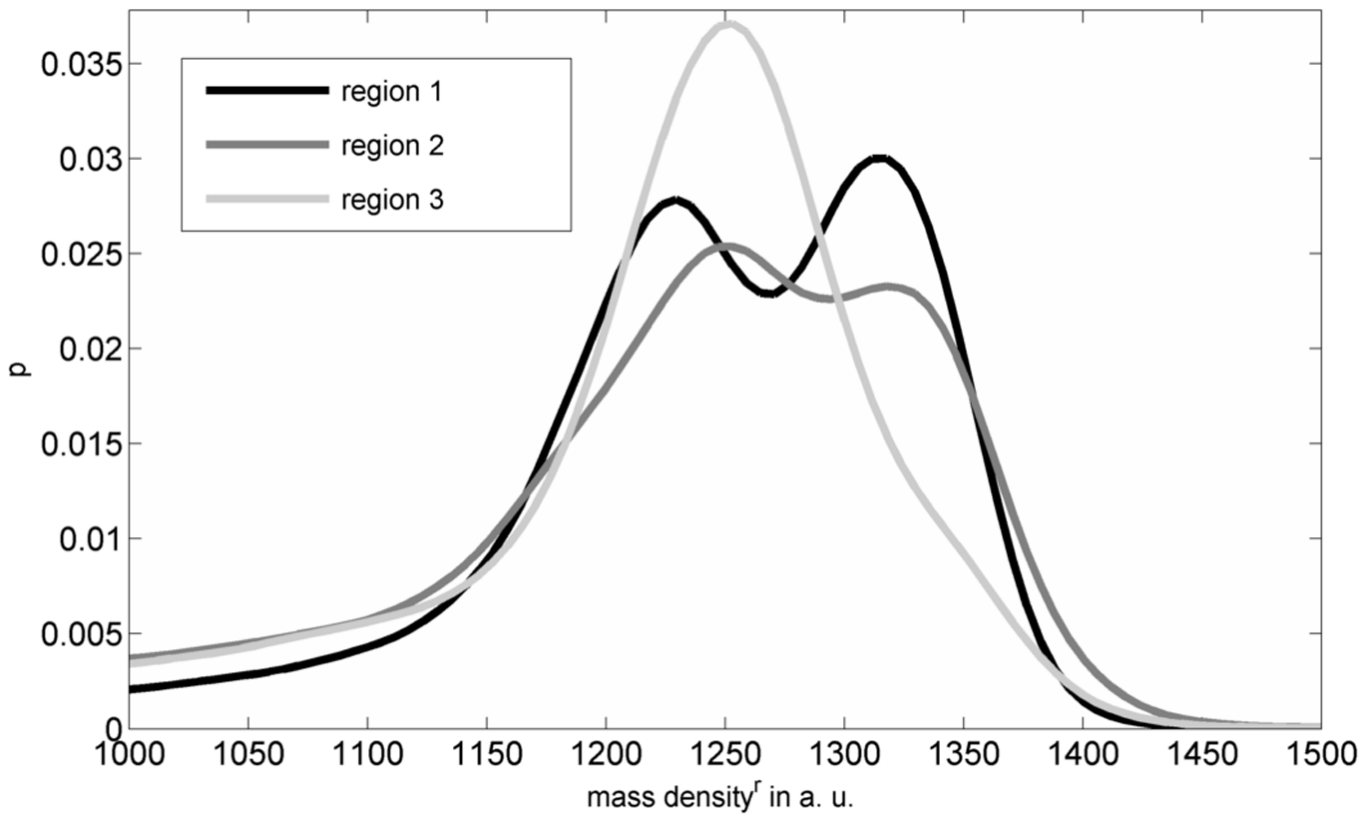
The 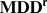 are shown for the three regions, with region 1, region 2, and region 3 being the top, the middle, and the lower sub-section of one specimen scanned, respectively.

Cross sectional µCT images and the corresponding segmented lacunar areas of a jaw bone sample are shown in Fig. 1. A 3D volume rendering of the osteocyte lacunae with subsections of one jaw and a femur sample image are shown in Fig. 3. These representative images exhibit distinct differences in the distribution and alignment of the lacunae in the different anatomical sites. The relative mass density histogram of the jaw specimen shown in Fig. 1 is shown in Fig. 2. The normalized histograms of the three adjacent sub-volumes of the same specimen in Fig. 4 illustrate the local intra-sample variability of 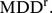


The average properties for the three different anatomical sites and the BRONJ samples are summarized in Table 2 and Table 3.

**Table 2:** Mean and standard deviation of the investigated properties for the different anatomical sites and pathology are summarized.

		Tibia	Femur	Jaw (control)	Jaw (BRONJ)
Number of ROI		9	21	27	29
Number of different donors		3	7	9	10
N.Lc		12530	11867	18665	15349
Lc.V_med_ in µm^3^		194 (27)	224 (42)	277 (117)	269 (80)
Lc.V_var_ in 1000 µm^6^		6.4 (1.9)	11.0 (3.6)	22.0 (11.5)	26.6 (8.9)
Lc.Dist_50_ in µm		11.1 (0.4)	15.2 (0.8)	12.9 (1.5)	13.9 (1.1)*
Lc.TV/BV in %		0.76 (0.09)	0.45 (0.09)	0.79 (0.32)	0.69 (0.17)
N.Lc/BV in 1000 mm^−3^		38 (5)	20 (2)	27 (6)	23 (4)*
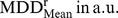		1301 (17)	1219 (29	1208 (66)	1225 (52)
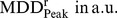		1322 (24)	1254 (68)	1277 (52)	1270 (48)
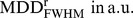		104 (38)	149 (72)	170 (66)	142 (38)
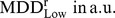		1194 (21)	1080 (33)	1048 (90)	1073 (73)
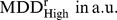		1408 (24)	1373 (33)	1360 (42)	1370 (45)

The * indicates that those properties are statistically significantly different between the BRONJ and control jaw.

**Table 3:** Mean and standard deviation of the investigated morphometric properties of the segmented vessel-pores for the different anatomical sites and pathology are summarized.

	Tibia	Femur	Jaw (control)	Jaw (BRONJ)
Ca.V/BV in %	5 (4)	8 (6)	7 (7)	7 (4)
Ca.S/Ca.V in µm^−1^	0.06 (0.04)	0.06 (0.03)	0.07 (0.04)	0.06 (0.02)
Ca.S/BV in µm^−1^	0.002 (0.001)	0.003 (0.001)	0.005 (0.004)	0.004 (0.002)

Ca.V: canal volume, BV: bone volume, Ca.S: canal surface.

### Differences between Anatomical Sites

**Figure 5:**
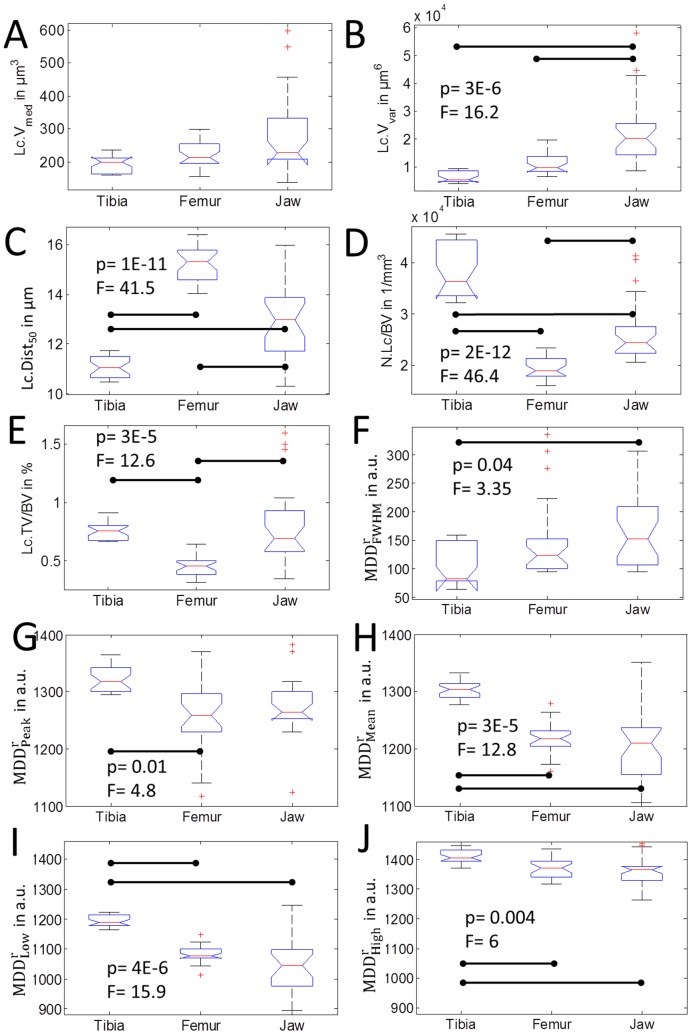
Investigated differences with respect to anatomical sites, assessed by analyses of variance (ANOVA), followed by post hoc multiple comparison Bonferroni tests are summarized. Significant differences between groups are indicated by a horizontal bar. If the significance level was reached, the p-Value and the F-Value are reported.

**Figure 6:**
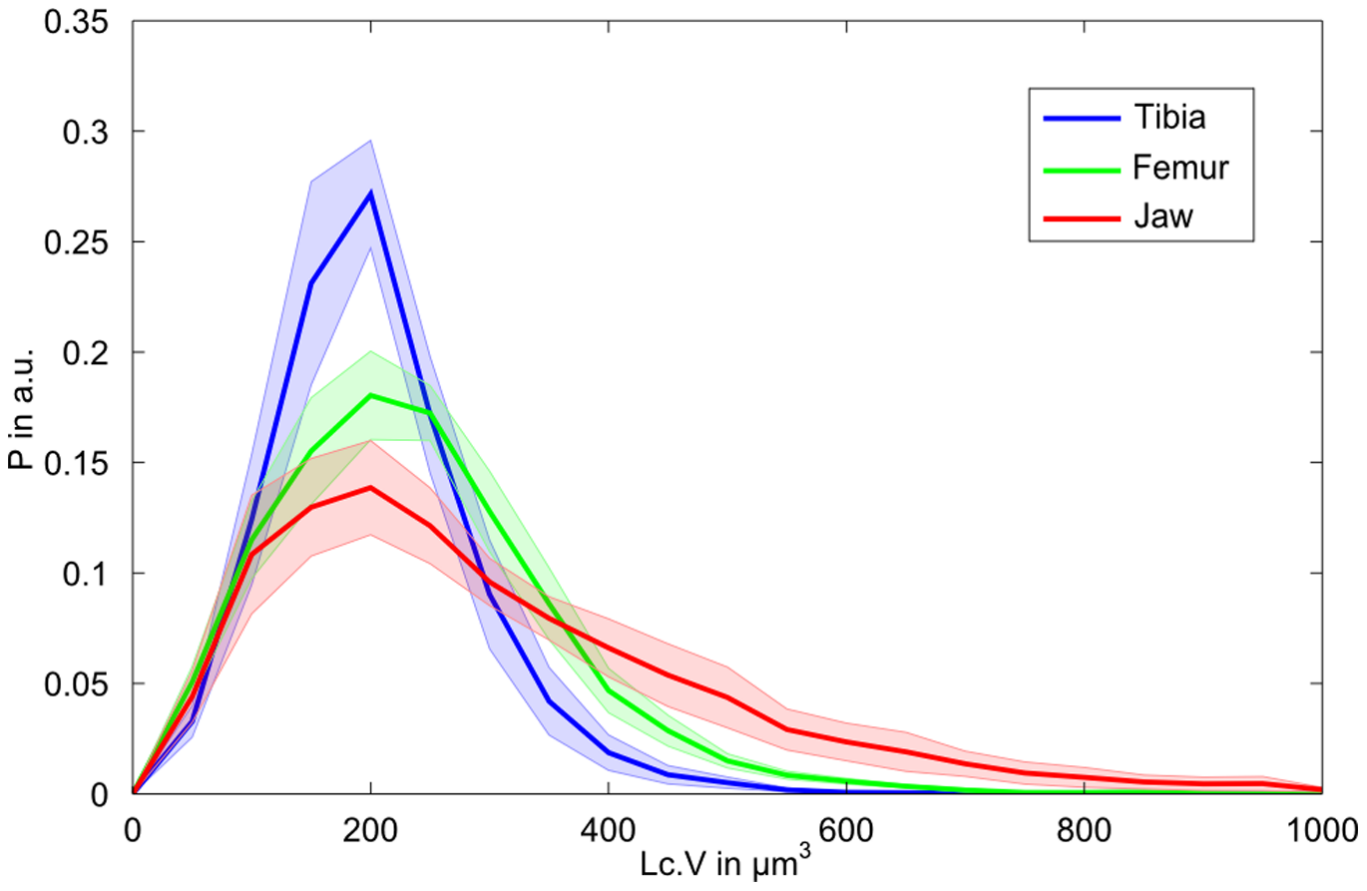
Histograms of the lacunar volumes for the three different sites are shown. Histograms are normalized to the area under the total number of lacunae for each site. Bin size is set to 50 µm^3^. The transparent areas indicate the standard error for each site based on the individual samples.

The average lacunar volume ranged from 194 µm^3^ in the tibia samples to 277 µm^3^ in the jaw bone. Both median values and variance (Figs. 5A–B) were significantly higher in the jaw bone compared to the other anatomical sites. Distinct distributions of lacunar volume between samples from the jaw bone and those obtained from the peripheral skeletal sites are also illustrated in Fig. 6, which shows comparable, almost normal distributions for tibia and femur samples, but a remarkable asymmetry towards high volumes in the jaw bone samples. The average distance, in which 50% of the tissue matrix with respect to the closest lacunae is located (Lc.Dist_50_), was highest in the femur, followed by the jaw bones and lowest in the tibiae (Fig. 5E). However, the standard deviation in jaw bone was considerably higher than in the other skeletal sites. For all investigated sites the average lacunar density (N.Lc/BV) was found to be larger than or equal to 20000 mm^−3^ (Fig. 5D). Variations between the anatomical sites reflected those observed for Lc.Dist_50_, i.e. the lacunar density was highest in the tibia and lowest in the femur. The lacunar density in the jaw bones was between those of the other two sites.

Mean, low, and high values of the relative mass density distribution were significantly higher in the tibia compared to jaw and femur (Figs. 5H–J). In contrast, the heterogeneity of 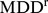 within the evaluated sub-volumes, as expressed by 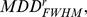 was higher in the jaw bone than in tibia samples.

The average and standard deviation of Ca.V/BV, Ca.S/BV and Ca.S/Ca.V of all sections were found to be (7±5) %, (0.004±0.003) µm^−1^, and (0.06±0.03) µm^−1^, respectively. All values are summarized in Table 3. ANOVA revealed no significant differences between the different anatomical sites or between jaw sections from healthy donors and BRONJ.

### Differences between BRONJ and Control Jaw Bones

**Figure 7:**
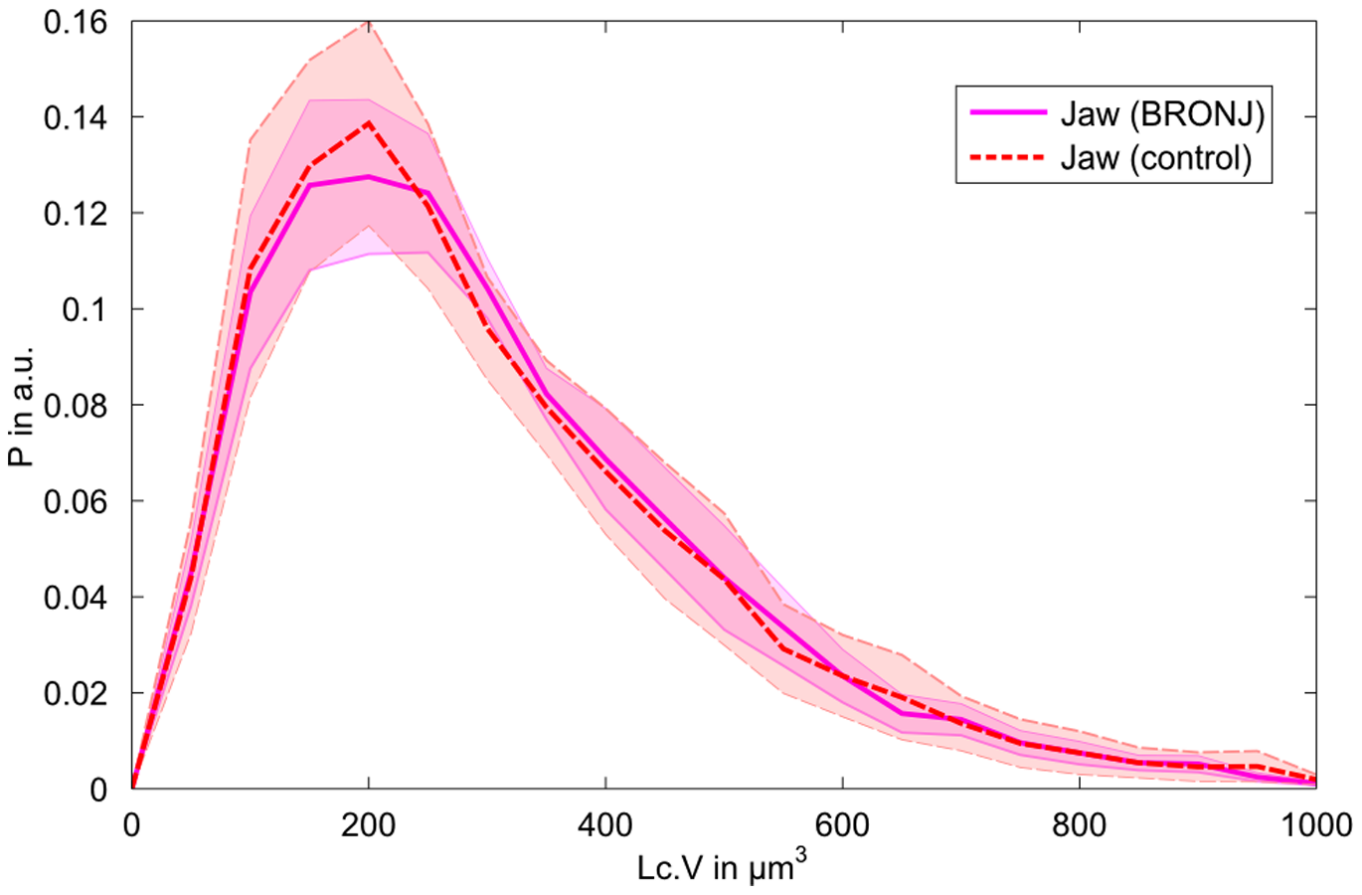
Histograms of all jaw lacunae grouped in either BRONJ or healthy bone. The shaded areas correspond to the standard error based on the different samples. Histograms are normalized to the absolute amount of lacunae, bin size is 50 µm^3^. It should be noted that even though the histograms of the two groups look very similar, there are differences between the histograms of individual donors.

Significantly lower lacunar densities (F = 5.1, p<.028) were observed in the BRONJ sample group (Table 2). The lower lacunar density was associated with higher Lc.Dist_50_ values (F = 6.7, p<.012). It should be noted that the values observed in the BRONJ samples are still within the range observed at other skeletal sites (see Table 2). All other evaluated parameters were statistically not significantly different when comparing BRONJ samples and healthy jaw bone controls. Most strikingly, the lacunar volume distributions of BRONJ and control jaw bone samples were almost identical (Fig. 7).

## Discussion

The present study aimed to determine the potential impact of alterations of the osteocyte lacunar network and mass density of the extracellular matrix, in the event of osteonecrosis after BP treatment. To address this question, we used synchrotron radiation phase micro-tomography with a 350 nm voxel size. We analyzed human cortical bone specimens extracted from the mandibular jaw of 10 patients suffering from BRONJ and of 9 healthy persons for control. In addition, we investigated samples from anatomical sites in which BP treatment does not usually induce necrosis, such as the tibia and femur.

The imaging technique used allows the investigation of relatively large sample volumes in 3D, without the necessity for demineralization or any other tissue preparation steps (except for ethanol fixation and drying), and combines a large field of view, a very high spatial resolution and a high signal-to-noise ratio with a good sensitivity to mass density fluctuations [52], [60]. Although the absolute mass density could not be derived, the derived relative mass density distributions 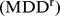 enabled a quantitative comparison between the evaluated samples.

Our results suggest that the osteocyte lacunar number, volume and distribution, as well as the mass density in the extracellular matrix, are closely linked to the anatomical site. While there are several studies reporting the human lacunar density based on 2D imaging modalities [61], [62] there are only a few reporting lacunar density in 3D, and these are from attenuation contrast tomography [43] at a lower resolution (1.47 µm voxel size) [40], [42]. The lacunar density values we found in the femoral samples are consistent with those we reported recently [43] and with those reported by Carter et al. [40] for the same anatomical region in women across their lifespan. In another study, significant variations were observed between anterior-posterior and medial-lateral regions with differences of up to 30% between the regions in single individuals, but no significant impact of age on N.Lc/BV [40]. Moreover, they found a significant decrease in the lacunar volume with respect to donor age (R^2^ = .46), whereas the lacunae from the younger group were ∼30% larger (age: <50 years) than those of the older group (age: >50 years). In contrast, no impact of age on lacunar size was reported by others [27].

We observed pronounced differences between jaw bones in comparison to the two peripheral skeletal bone sites. This may be explained by the different origin of the cells and the different remodeling rates in those tissues, or the different mechanical environment. While osteocytes in the peripheral skeleton derive from the trunk lateral plate mesoderm, in the mandible they derive from the paraxial mesoderm [63], [64]. The remodeling rate in jaw bone is believed to be higher than in femur and tibia [11], [65], which leads to lower mineralization and mass density [12] in the jaw. The latter has been confirmed only partly in our study by means of lower distribution values of 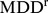 (mean, low and high) and a higher intra-specimen variability (in terms of FWHM) in the jaw bones compared to tibia (Fig. 5F–J). However, we observe a trend towards smaller values for jaw compared to femur (mean, low and high), which is in line with the lower remodeling rate in the femur reported earlier [66].

The majority of the osteocyte lacunar volumes were in the range between 100 and 400 µm^3^, which is in agreement with values reported in other studies [40], [42], [43]. However, a small fraction of very large osteocyte lacunae with volumes between 400 and 1000 µm^3^ was found in the jaw bone samples (Fig. 6). Most strikingly, this characteristic asymmetric distribution of osteocyte lacunar volume was almost identical in healthy jaw bone samples and those treated with BP (Fig. 7). The only significant effect of the BP treatment in jaw bone appeared to be the reduction of lacunar density, which was also evident in the increased average distance between the extracellular matrix and the closest osteocyte lacuna. On average, the samples treated with BP had a lacunar density 14.8% lower than the healthy jaw bone controls, but still 15% higher than the femur samples. Mass density (mean, low, and high values) was not significantly higher in the jaws treated with BP in comparison to the healthy controls, although these values were marginally higher and the heterogeneity (FWHM) was slightly lower than those in the control group. Therefore, it must be concluded that no remarkable changes of mass density occur in the extracellular matrix after BP treatment. For comparison, the mass density distribution values of the jaw bones treated with BP do not reach the values observed in tibia and femur samples. However, with the current analysis we cannot exclude potential local changes, for example directly at the pore tissue boundary. It is well accepted that osteocytes actively remodel their direct extracellular environment [35] and it has been shown by fluorescence-labeled risedronate injected intravenously in a murine model that BP is indeed deposited around the osteocyte lacunae [67]. Interestingly, the results of that study have suggested that the deposition rate was not uniform in all osteocytes, but decreased along with the distance to the next vascular channel. Therefore, a dedicated regional analysis of the lacunar-matrix interface merits further investigation. Overall, except for the reduction in osteocyte lacunar number, the changes of the lacunar network and the mass density of the extracellular matrix appeared marginal. In particular, the BP-induced parameter alterations in the jaw stayed within the variations observed between different anatomical sites. Consequently, the observed parameter variations are not likely to be the primary causes for the development of BRONJ in the jaw.

In this study, we have not investigated the lacunar occupation rate and viability of osteocytes, as the increased number of abandoned osteocyte lacunae is a well-documented sign of BP treatment in the jaw [64], [68]. Moreover, the filling of empty lacunae with mineral has been reported in several studies [69]–[71], which is in line with the reduced lacunar density we observed in the jaw bones treated with BP. It is known that BPs have a high affinity to hydroxyapatite [63]. Therefore, we hypothesize that higher perfusion and turnover rates initially lead to a higher deposition of BP in the human jaw compared to other human sites, which is in line with the findings from previous studies in animals [11]. At higher doses BP becomes cytotoxic [72], which presumably promotes the gradual depletion of osteocytes and allows the abandoned pores to be filled with more BP-loaded mineral. Finally, the acidification of this tissue, caused for example by an inflammation, which is known to occur in BRONJ, can result in an excessive dissolution of BP-loaded mineral and a release of toxic doses of BP. Among the multifold biological factors promoting the development of necrosis in the jaw, the fraction of large lacunae (>400 µm^3^) found in jaw bone tissue but not in the tissues from the other skeletal sites may be a structural factor, since it allows the deposition of larger amounts of highly concentrated BP-loaded mineral in such abandoned lacunae, which when washed out results in higher and thus more toxic local BP doses.

We did not observe significant differences in vessel porosities between the different groups, which supports the idea that the observed differences in lacunar properties and mass density distributions between the different groups are not linked to the potential differences in vessel porosities in the investigated specimens. The reported pore-volume ratios are within the range previously shown for femoral cortical bone [43]. However, the quantification of vessel porosities was limited in our study, due to the size of the field of view, which was small in comparison to the average distance between individual vessels. Therefore, the field of view cannot be considered to be a representative volume with respect to the vessel network.

This study has several limitations. One drawback of the present study is that the comparison between sites and even within the jaw bone is hampered by our limited ability to control the exact anatomical location and orientation of the harvested samples and the underlying diagnosis. Moreover, the samples from femur, tibia, and jaw bones could not be collected from the same donors, and the duration and type of BP treatment was not uniform within the BRONJ group. Potential influencing factors, such as intra-specimen variability, age and gender, as well as the BP treatment conditions, could have biased our analysis. Additionally, in contrast to previous investigations in which we have demonstrated the feasibility to extract absolute mass density values from the phase contrast images [46], [47], we report relative values in this study. This is due to the fact that (i) the large distance between sample and detector in the current configuration violated the near-field condition, which is an essential prerequisite for the Paganin-phase retrieval used [58] and (ii) we used a constant ratio of delta over beta for the Paganin retrieval, even though it could have been different between samples with different degrees of mineralization [73].

Additionally, since we observed a bimodal distribution of the apparent mass densities (Fig. 4), and the samples were comparable small in terms of being representative for the ratio of interstitial to osteonal tissue, 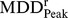 may not be an appropriate parameter to quantify 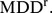


Nevertheless, the subtle ultra-structural alterations observed in the jaws treated with BP underline the need for further sophisticated investigations of large tissue volume with sub-micron resolution, high sensitivity to local changes in mineral density and chemical composition of the tissue. Such changes are not likely to be depicted by conventional X-ray methods. Phase contrast tomography with voxel sizes ranging down to about 50 nm is now available at SR sources and may provide new hints towards the ultra-structural mechanisms leading to the pathogenesis of BRONJ.

## References

[1] Dhillon S, Lyseng-Williamson KA (2008) Zoledronic acid - A review of its use in the management of bone metastases of malignancy. Drugs 68: 507–534. 18318568 10.2165/00003495-200868040-00010

[2] Liberman UA, Weiss SR, Broll J, Minne HW, Quan H, et al (1995) Effect of Oral Alendronate on Bone-Mineral Density and the Incidence of Fractures in Postmenopausal Osteoporosis. New England Journal of Medicine 333: 1437–1443. 7477143 10.1056/NEJM199511303332201

[3] (2009) Chmp Assessment Report on Bisphosphonates and Osteonecrosis of the Jaw. European Medicines Agency.

[4] Ruggiero SL, Dodson TB, Assael LA, Landesberg R, Marx RE, et al (2009) American Association of Oral and Maxillofacial Surgeons Position Paper on Bisphosphonate-Related Osteonecrosis of the Jaws–2009 Update. Journal of Oral and Maxillofacial Surgery 67: 2–12. 10.1016/j.joms.2009.01.009 19371809

[5] Mercer E, Norton T, Woo S, Treister N, Dodson TB, et al. (2013) Ninety-One Osteoporosis Patients Affected with Bisphosphonate-Related Osteonecrosis of the Jaw: A Case Series. Calcif Tissue Int. 10.1007/s00223-013-9747-1 PMC3744621 23756612

[6] Kuhl S, Walter C, Acham S, Pfeffer R, Lambrecht JT (2012) Bisphosphonate-related osteonecrosis of the jaws–a review. Oral Oncol 48: 938–947. 22525606 10.1016/j.oraloncology.2012.03.028

[7] Allen MR, Ruggiero SL (2009) Higher bone matrix density exists in only a subset of patients with bisphosphonate-related osteonecrosis of the jaw. J Oral Maxillofac Surg 67: 1373–1377. 19531405 10.1016/j.joms.2009.03.048

[8] Lesclous P, Abi Najm S, Carrel JP, Baroukh B, Lombardi T, et al (2009) Bisphosphonate-associated osteonecrosis of the jaw: a key role of inflammation? Bone 45: 843–852. 19631301 10.1016/j.bone.2009.07.011

[9] Otto S, Hafner S, Mast G, Tischer T, Volkmer E, et al (2010) Bisphosphonate-related osteonecrosis of the jaw: is pH the missing part in the pathogenesis puzzle? J Oral Maxillofac Surg 68: 1158–1161. 20138420 10.1016/j.joms.2009.07.079

[10] Meiller T, Almubarak H, Weikel D, Brahim J, Scheper M (2012) Bisphosphonate-associated osteonecrosis of the jaw: are we dealing with a localized non-traditional calciphylaxis? Open Dent J 6: 5–7. 22276079 10.2174/1874210601206010005 PMC3263445

[11] Bertoldo F, Santini D, Lo Cascio V (2007) Bisphosphonates and osteomyelitis of the jaw: a pathogenic puzzle. Nat Clin Pract Oncol 4: 711–721. 18037875 10.1038/ncponc1000

[12] Ruffoni D, Fratzl P, Roschger P, Klaushofer K, Weinkamer R (2007) The bone mineralization density distribution as a fingerprint of the mineralization process. Bone 40: 1308–1319. 17337263 10.1016/j.bone.2007.01.012

[13] Roschger P, Paschalis EP, Fratzl P, Klaushofer K (2008) Bone mineralization density distribution in health and disease. Bone 42: 456–466. 18096457 10.1016/j.bone.2007.10.021

[14] Bach-Gansmo FL, Irvine SC, Bruel A, Thomsen JS, Birkedal H (2013) Calcified cartilage islands in rat cortical bone. Calcif Tissue Int 92: 330–338. 23274728 10.1007/s00223-012-9682-6

[15] Boivin G, Meunier PJ (2002) The degree of mineralization of bone tissue measured by computerized quantitative contact microradiography. Calcif Tissue Int 70: 503–511. 12019458 10.1007/s00223-001-2048-0

[16] Nuzzo S, Lafage-Proust MH, Martin-Badosa E, Boivin G, Thomas T, et al (2002) Synchrotron radiation microtomography allows the analysis of three-dimensional microarchitecture and degree of mineralization of human iliac crest biopsy specimens: effects of etidronate treatment. J Bone Miner Res 17: 1372–1382. 12162491 10.1359/jbmr.2002.17.8.1372

[17] Currey JD (1984) Effects of differences in mineralization on the mechanical properties of bone. Philos Trans R Soc Lond B Biol Sci 304: 509–518. 6142490 10.1098/rstb.1984.0042

[18] Bonewald LF (2011) The amazing osteocyte. J Bone Miner Res 26: 229–238. 21254230 10.1002/jbmr.320 PMC3179345

[19] Marotti G, Ferretti M, Remaggi F, Palumbo C (1995) Quantitative evaluation on osteocyte canalicular density in human secondary osteons. Bone 16: 125–128. 7742070 10.1016/s8756-3282(94)00019-0

[20] Klein-Nulend J, Bakker AD, Bacabac RG, Vatsa A, Weinbaum S (2013) Mechanosensation and transduction in osteocytes. Bone 54: 182–190. 23085083 10.1016/j.bone.2012.10.013

[21] Schneider P, Meier M, Wepf R, Muller R (2010) Towards quantitative 3D imaging of the osteocyte lacuno-canalicular network. Bone 47: 848–858. 20691297 10.1016/j.bone.2010.07.026

[22] Lanyon LE (1993) Osteocytes, strain detection, bone modeling and remodeling. Calcif Tissue Int 53 Suppl 1S102–106 discussion S106–107. 8275362 10.1007/BF01673415

[23] Burger EH, Klein-Nulend J (1999) Mechanotransduction in bone–role of the lacuno-canalicular network. FASEB J 13 Suppl: S101–112 10352151

[24] Zhou X, Novotny JE, Wang L (2009) Anatomic variations of the lacunar-canalicular system influence solute transport in bone. Bone 45: 704–710. 19576310 10.1016/j.bone.2009.06.026 PMC2730977

[25] Wang N, Butler JP, Ingber DE (1993) Mechanotransduction across the cell surface and through the cytoskeleton. Science 260: 1124–1127. 7684161 10.1126/science.7684161

[26] Weinbaum S, Cowin SC, Zeng Y (1994) A model for the excitation of osteocytes by mechanical loading-induced bone fluid shear stresses. J Biomech 27: 339–360. 8051194 10.1016/0021-9290(94)90010-8

[27] McCreadie BR, Hollister SJ, Schaffler MB, Goldstein SA (2004) Osteocyte lacuna size and shape in women with and without osteoporotic fracture. J Biomech 37: 563–572. 14996569 10.1016/S0021-9290(03)00287-2

[28] Vatsa A, Breuls RG, Semeins CM, Salmon PL, Smit TH, et al (2008) Osteocyte morphology in fibula and calvaria – is there a role for mechanosensing? Bone 43: 452–458. 18625577 10.1016/j.bone.2008.01.030

[29] van Hove RP, Nolte PA, Vatsa A, Semeins CM, Salmon PL, et al (2009) Osteocyte morphology in human tibiae of different bone pathologies with different bone mineral density–is there a role for mechanosensing? Bone 45: 321–329. 19398046 10.1016/j.bone.2009.04.238

[30] Currey JD (2003) The many adaptations of bone. J Biomech 36: 1487–1495. 14499297 10.1016/s0021-9290(03)00124-6

[31] Mullins LP, McGarry JP, Bruzzi MS, McHugh PE (2007) Micromechanical modelling of cortical bone. Comput Methods Biomech Biomed Engin 10: 159–169. 17558645 10.1080/10255840601110802

[32] Currey JD (1984) The Mechanical Adaption of Bone. Princeton University Press.

[33] Westbroek I, De Rooij KE, Nijweide PJ (2002) Osteocyte-specific monoclonal antibody MAb OB7.3 is directed against Phex protein. J Bone Miner Res 17: 845–853. 12009015 10.1359/jbmr.2002.17.5.845

[34] Nakashima T, Hayashi M, Fukunaga T, Kurata K, Oh-Hora M, et al (2011) Evidence for osteocyte regulation of bone homeostasis through RANKL expression. Nat Med 17: 1231–1234. 21909105 10.1038/nm.2452

[35] Qing H, Ardeshirpour L, Pajevic PD, Dusevich V, Jahn K, et al (2012) Demonstration of osteocytic perilacunar/canalicular remodeling in mice during lactation. J Bone Miner Res 27: 1018–1029. 22308018 10.1002/jbmr.1567 PMC3770147

[36] Blaber EA, Dvorochkin N, Lee C, Alwood JS, Yousuf R, et al (2013) Microgravity induces pelvic bone loss through osteoclastic activity, osteocytic osteolysis, and osteoblastic cell cycle inhibition by CDKN1a/p21. PLoS One 8: e61372. 23637819 10.1371/journal.pone.0061372 PMC3630201

[37] Lane NE, Yao W, Balooch M, Nalla RK, Balooch G, et al (2006) Glucocorticoid-treated mice have localized changes in trabecular bone material properties and osteocyte lacunar size that are not observed in placebo-treated or estrogen-deficient mice. J Bone Miner Res 21: 466–476. 16491295 10.1359/JBMR.051103 PMC1797152

[38] Tommasini SM, Trinward A, Acerbo AS, De Carlo F, Miller LM, et al (2012) Changes in intracortical microporosities induced by pharmaceutical treatment of osteoporosis as detected by high resolution micro-CT. Bone 50: 596–604. 22226688 10.1016/j.bone.2011.12.012 PMC3278519

[39] Webster DJ, Schneider P, Dallas SL, Muller R (2013) Studying osteocytes within their environment. Bone. 10.1016/j.bone.2013.01.004 PMC3652555 23318973

[40] Carter Y, Thomas CD, Clement JG, Peele AG, Hannah K, et al (2013) Variation in osteocyte lacunar morphology and density in the human femur–a synchrotron radiation micro-CT study. Bone 52: 126–132. 22995461 10.1016/j.bone.2012.09.010

[41] Dong P, Haupert S, Gouttenoire P-J, Peyrin F (2013) Efficient extraction of 3D bone cells descriptors from micro-CT images; 2013 7–11 1492–1495.

[42] Carter Y, Thomas CD, Clement JG, Cooper DM (2013) Femoral osteocyte lacunar density, volume and morphology in women across the lifespan. J Struct Biol 183: 519–526. 23872433 10.1016/j.jsb.2013.07.004

[43] Dong P, Haupert S, Hesse B, Langer M, Gouttenoire P-J, et al (2013) 3D osteocyte lacunar morphometric properties and distributions in human femoral cortical bone using synchrotron radiation micro-CT images. Bone 60: 172–185. 24334189 10.1016/j.bone.2013.12.008

[44] Pacureanu A, Langer M, Boller E, Tafforeau P, Peyrin F (2012) Nanoscale imaging of the bone cell network with synchrotron X-ray tomography: optimization of acquisition setup. Med Phys 39: 2229–2238. 22482644 10.1118/1.3697525

[45] Dierolf M, Menzel A, Thibault P, Schneider P, Kewish CM, et al (2010) Ptychographic X-ray computed tomography at the nanoscale. Nature 467: 436–439. 20864997 10.1038/nature09419

[46] Langer M, Pacureanu A, Suhonen H, Grimal Q, Cloetens P, et al (2012) X-ray phase nanotomography resolves the 3D human bone ultrastructure. PLoS One 7: e35691. 22952569 10.1371/journal.pone.0035691 PMC3430646

[47] Varga P, Pacureanu A, Langer M, Suhonen H, Hesse B, et al (2013) Investigation of the 3D orientation of mineralized collagen fibrils in human lamellar bone using synchrotron X-ray phase nano-tomography. Acta Biomater 9: 8118–8127. 23707503 10.1016/j.actbio.2013.05.015

[48] Kingsmill VJ, Boyde A (1998) Mineralisation density of human mandibular bone: quantitative backscattered electron image analysis. J Anat 192 (Pt 2): 245–256. 10.1046/j.1469-7580.1998.19220245.x PMC1467758 9643425

[49] Dall’Ara E, Luisier B, Schmidt R, Kainberger F, Zysset P, et al (2013) A nonlinear QCT-based finite element model validation study for the human femur tested in two configurations in vitro. Bone 52: 27–38. 22985891 10.1016/j.bone.2012.09.006

[50] Kazakia GJ, Burghardt AJ, Cheung S, Majumdar S (2008) Assessment of bone tissue mineralization by conventional x-ray microcomputed tomography: comparison with synchrotron radiation microcomputed tomography and ash measurements. Med Phys 35: 3170–3179. 18697542 10.1118/1.2924210 PMC2673562

[51] Lakshmanan S, Bodi A, Raum K (2007) Assessment of Anisotropic Tissue Elasticity of Cortical Bone from High-Resolution, Angular Acoustic Measurements. Ultrasonics, Ferroelectrics and Frequency Control, IEEE Transactions on 54: 1560–1570. 10.1109/tuffc.2007.426 17703659

[52] Marinescu M, Langer M, Durand A, Olivier C, Chabrol A, et al. (2013) Synchrotron Radiation X-Ray Phase Micro-computed Tomography as a New Method to Detect Iron Oxide Nanoparticles in the Brain. Mol Imaging Biol. 10.1007/s11307-013-0639-6 23632952

[53] Cloetens P, Pateyron-Salome M, Buffiere JY, Peix G, Baruchel J, et al (1997) Observation of microstructure and damage in materials by phase sensitive radiography and tomography. Journal of Applied Physics 81: 5878–5886.

[54] Paganin D, Mayo SC, Gureyev TE, Miller PR, Wilkins SW (2002) Simultaneous phase and amplitude extraction from a single defocused image of a homogeneous object. J Microsc 206: 33–40. 12000561 10.1046/j.1365-2818.2002.01010.x

[55] del Río MS, Dejus RJ (2004) XOP 2.1 – A New Version of the X-ray Optics Software Toolkit. AIP Conference Proceedings 705: 784–787.

[56] Raum K, Cleveland RO, Peyrin F, Laugier P (2006) Derivation of elastic stiffness from site-matched mineral density and acoustic impedance maps. Phys Med Biol 51: 747–758. 16424593 10.1088/0031-9155/51/3/018

[57] Yoo TS, Ackerman MJ, Lorensen WE, Schroeder W, Chalana V, et al (2002) Engineering and algorithm design for an image processing Api: a technical report on ITK–the Insight Toolkit. Stud Health Technol Inform 85: 586–592. 15458157

[58] Weitkamp T, Haas D, Wegrzynek D, Rack A (2011) ANKAphase: software for single-distance phase retrieval from inline X-ray phase-contrast radiographs. J Synchrotron Radiat 18: 617–629. 21685680 10.1107/S0909049511002895

[59] Jarque CM, Bera AK (1987) A test for normality of observations and regression residuals. International Statistical Review Vol. 55: 163–172.

[60] Diemoz PC, Bravin A, Langer M, Coan P (2012) Analytical and experimental determination of signal-to-noise ratio and figure of merit in three phase-contrast imaging techniques. Opt Express 20: 27670–27690. 23262715 10.1364/OE.20.027670

[61] Qiu S, Rao DS, Palnitkar S, Parfitt AM (2006) Differences in osteocyte and lacunar density between Black and White American women. Bone 38: 130–135. 16112633 10.1016/j.bone.2005.07.004

[62] Mullender MG, Tan SD, Vico L, Alexandre C, Klein-Nulend J (2005) Differences in osteocyte density and bone histomorphometry between men and women and between healthy and osteoporotic subjects. Calcif Tissue Int 77: 291–296. 16307389 10.1007/s00223-005-0043-6

[63] Russell RGG, Watts NB, Ebetino FH, Rogers MJ (2008) Mechanisms of action of bisphosphonates: similarities and differences and their potential influence on clinical efficacy. Osteoporosis International 19: 733–759. 18214569 10.1007/s00198-007-0540-8

[64] Bonnet N, Lesclous P, Saffar JL, Ferrari S (2013) Zoledronate effects on systemic and jaw osteopenias in ovariectomized periostin-deficient mice. PLoS One 8: e58726. 23505553 10.1371/journal.pone.0058726 PMC3591374

[65] Yu YY, Lieu S, Hu D, Miclau T, Colnot C (2012) Site Specific Effects of Zoledronic Acid during Tibial and Mandibular Fracture Repair. PLoS One 7: e31771. 22359627 10.1371/journal.pone.0031771 PMC3281002

[66] Huja SS, Fernandez SA, Hill KJ, Li Y (2006) Remodeling dynamics in the alveolar process in skeletally mature dogs. Anat Rec A Discov Mol Cell Evol Biol 288: 1243–1249. 17075846 10.1002/ar.a.20396 PMC2612758

[67] Roelofs AJ, Coxon FP, Ebetino FH, Lundy MW, Henneman ZJ, et al (2010) Fluorescent risedronate analogues reveal bisphosphonate uptake by bone marrow monocytes and localization around osteocytes in vivo. J Bone Miner Res 25: 606–616. 20422624 10.1359/jbmr.091009 PMC3153397

[68] Maurer P, Sandulescu T, Kriwalsky MS, Rashad A, Hollstein S, et al (2011) Bisphosphonate-related osteonecrosis of the maxilla and sinusitis maxillaris. Int J Oral Maxillofac Surg 40: 285–291. 21163624 10.1016/j.ijom.2010.11.006

[69] Frost HM (1960) Micropetrosis. J Bone Joint Surg Am 42-A: 144–150. 13849862

[70] Carpentier VT, Wong J, Yeap Y, Gan C, Sutton-Smith P, et al (2012) Increased proportion of hypermineralized osteocyte lacunae in osteoporotic and osteoarthritic human trabecular bone: implications for bone remodeling. Bone 50: 688–694. 22173055 10.1016/j.bone.2011.11.021

[71] Busse B, Djonic D, Milovanovic P, Hahn M, Puschel K, et al (2010) Decrease in the osteocyte lacunar density accompanied by hypermineralized lacunar occlusion reveals failure and delay of remodeling in aged human bone. Aging Cell 9: 1065–1075. 20874757 10.1111/j.1474-9726.2010.00633.x

[72] Allen MR, Burr DB (2008) Mandible matrix necrosis in beagle dogs after 3 years of daily oral bisphosphonate treatment. J Oral Maxillofac Surg 66: 987–994. 18423290 10.1016/j.joms.2008.01.038 PMC2464292

[73] Langer M, Cloetens P, Hesse B, Suhonen H, Pacureanu A, et al. (2014) Priors for X-ray in-line phase tomography of heterogeneous objects. Phil Trans R Soc A: In press. 10.1098/rsta.2013.0129 24470421

